# Comparison of warfarin, rivaroxaban, and dabigatran for effectiveness and safety in atrial fibrillation patients with different CHA2DS2-VASc scores: a retrospective cohort study

**DOI:** 10.1186/s12872-024-04020-9

**Published:** 2024-07-16

**Authors:** Yue Zhao, Hong Ren, Shiwei Xu

**Affiliations:** 1 Department of Pharmacy, the Second Affiliated Hospital of Harbin Medical University, Harbin, 150086 P.R. China; 2 Department of Pharmacy Intravenous Admixture Service, the First Affiliated Hospital of Harbin Medical University, Harbin, 150001 P.R. China

**Keywords:** CHA2DS2-VASc score, Warfarin, Rivaroxaban, Dabigatran, Atrial fibrillation

## Abstract

**Background:**

This retrospective cohort study aims to compare the effectiveness and safety of warfarin, rivaroxaban, and dabigatran in atrial fibrillation (AF) patients with different CHA2DS2-VASc scores in northern China.

**Methods:**

A retrospective cohort study was performed to evaluate anticoagulation in AF patients at the second affiliated hospital of Harbin Medical University from September 2018 to August 2019. Patients included in this study (*n* = 806) received warfarin (*n* = 300), or rivaroxaban (*n* = 203), or dabigatran (*n* = 303). Baseline characteristics and follow-up data including adherence, bleeding events and ischemic stroke (IS) events were collected.

**Results:**

Patients receiving rivaroxaban (73.9%) or dabigatran (73.6%) showed better adherence than those receiving warfarin (56.7%). Compared with warfarin-treated patients, dabigatran-treated patients had lower incidence of bleeding events (10.9% vs 19.3%, χ^2^ = 8.385, *P* = 0.004) and rivaroxaban-treated patients had lower incidence of major adverse cardiovascular events (7.4% vs 13.7%, χ^2^ = 4.822, *P* = 0.028). We classified patients into three groups based on CHA2DS2-VASc score (0–1, 2–3, ≥ 4). In dabigatran intervention, incidence of bleeding events was higher in patients with score 0–1 (20.0%) than those with score 2–3 (7.9%, χ^2^ = 5.772, *P* = 0.016) or score ≥ 4 (8.6%, χ^2^ = 4.682, *P* = 0.030). Patients with score 0–1 in warfarin or rivaroxaban therapy had a similar but not significant increase of bleeding compared with patients with score 2–3 or score ≥ 4, respectively. During the follow-up, 33 of 806 patients experienced IS and more than half (19, 57.6%) were patients with score ≥ 4. Comparing patients with score 0–1 and 2–3, the latter had an significant reduction of IS in patients prescribed warfarin and non-significant reduction in rivaroxaban and dabigatran therapy.

**Conclusion:**

Compared with warfarin therapy, patients with different CHA2DS2-VASc scores receiving either rivaroxaban or dabigatran were associated with higher persistence. AF patients with score ≥ 4 were more likely to experience IS events while hemorrhagic tendency preferred patients with low score 0–1.

## Introduction

Atrial fibrillation (AF) is the most common sustained cardiac arrhythmia with estimated prevalence between 2% and 4% in adults. AF poses great burden to patients, societal health and health economy. Ischemic stroke (IS) is the major complication of AF. Most patients with AF require appropriate anticoagulation to reduce IS events and all-cause mortality [1–3]. In northern China, due to specific risk factors including high-salt diet and cold weather induced hypertension, incidence of IS and AF is high and special attention is needed [4].

Oral anticoagulants (OACs) including Vitamin K antagonists (VKA, mostly warfarin) and non-vitamin K antagonist oral anticoagulants (NOACs, rivaroxaban, dabigatran, edoxaban and apixaban) are main stroke prevention medications for AF patients [5, 6]. In our hospital, apixaban is not available, so patients receiving NOACs take either rivaroxaban or dibigatran. Both VKA and NOACs can reduce stroke related to AF and all-cause mortality. According to meta-analyses, VKA can reduce stroke or systemic embolism by 64% compared with placebo or controls and NOACs induce a reduction of 19% compared with warfarin [7]. When it comes to choosing anticoagulants, VKA and NOACs have their own advantages and disadvantages. Warfarin is the only anticoagulants recommended by guidelines for AF patients with moderate or severe rheumatic mitral valve diseases and mechanical heart valve replacement [8]. Cost effectiveness is an important factor that physicians must take into consideration. Compared with NOACs, warfarin is more affordable with some patients due to its friendly price. Limitations of warfarin include narrow therapeutic range, necessitating frequent monitoring international normalized ratio (INR) and dose adjustments. Persistence to NOACs is generally higher than to warfarin, mainly due to no requirement of frequent monitoring [9, 10].

While AF increases five-fold risk of stroke, not all AF patients experience stroke. It’s depending on presence of specific risk factors. Most AF guidelines recommend assessing stroke risk before initiating antithrombotic therapy or not according to CHA2DS2-VASc [Congestive heart failure, Hypertension, Age ≥ 75, Diabetes mellitus, Stroke, Vascular disease, Age 65–74, Sex category (female)] score and physicians’ discretion [11, 12]. For instance, 2020 Europe Society of Cardiology guidelines for the diagnosis and management of AF recommend: for AF patients with score 0 in males or 1 in females with low stroke risk, no antithrombotic treatment is recommended; 1 in males or 2 in females, OAC should be considered; ≥ 2 in males or ≥ 3 in females, OAC is recommended [1]. Although CHA2DS2-VASc score can assess IS risk of AF patients, limited real-world study on northern China population is available on the safety and efficacy of different anticoagulants in patients with different scores. In this retrospective, single-center cohort study, we collected baseline characteristics and follow-up data including adherence, bleeding events and IS events to compare the effectiveness and safety of warfarin, rivaroxaban, and dabigatran in northern Chinese AF patients with different CHA2DS2-VASc scores.

## Methods

### Study design

All participants were AF patients discharged from the Second Affiliated Hospital of Harbin Medical University (Harbin, China) between September 2018 and August 2019. We included AF patients (> 18 years of age) receiving warfarin (300 patients, 0.625—6.25 mg/d, INR 2.0—3.0, TTR > 80%), or rivaroxaban (203 patients, 10 mg/d, 15 mg/d or 20 mg/d), or dabigatran (303 patients, 110 mg or 150 mg twice daily). Patients with rheumatic mitral stenosis, mitral valve repair, mechanical valve replacement or switched anticoagulants were excluded. The follow-up period was 6—12 months. The study was approved by the ethics committee of the Second Affiliated Hospital of Harbin Medical University (KY 2020–195).

### Baseline characteristics

The following demographic and clinical baseline characteristics were collected from medical records: age, gender, history of smoking or drinking, comorbidities (heart failure, hypertension, diabetes mellitus, history of stroke, vascular diseases, liver or renal diseases), experimental indicators including creatinine, low-density lipoprotein cholesterol-C (LDL-C), high-density lipoprotein cholesterol-C (HDL-C), total cholesterol, triglyceride, lipoprotein A and B, uric acid, left atrial diameter (LAD), left ventricular ejection fraction (LVEF). CHA2DS2-VASc scores were calculated accordingly.

### Patients follow-up

Clinical information of all participants were collected from hospitalization records, outpatient medical records and telephone questionaires. The median follow-up time was 11 month for warfarin group, 10 month for rivaroxaban group and 10 month for dibigatran group. During the follow-up period, information such as patients’ clinical condition, outcomes, medication persistence and adverse effects were collected and analyzed.

### Study outcomes

Outcome measures of safety were bleeding incidents (hemorrhinia, fundus hemorrhage, gingival bleeding and gastrointestinal bleeding). Outcome measures of effectiveness were incidence of IS and major adverse cardiovascular events (MACE, defined death, myocardial infarction or stroke) were also evaluated.

### Statistical analysis

Patients were divided into three groups according to CHA2DS2-VASc score (0–1, 2–3 and ≥ 4). Data are shown as numbers and percent for categorical variables and mean ± SEM for continuous variables. Chi-square test or Fisher’s exact test was used to evaluate categorical variables and Students *t* test for continuous data. Statistical analyses were performed by SPSS 25 (SPSS, USA). *P* < 0.05 was considered statistically significant.

## Results

### Study population

**Fig. 1:**
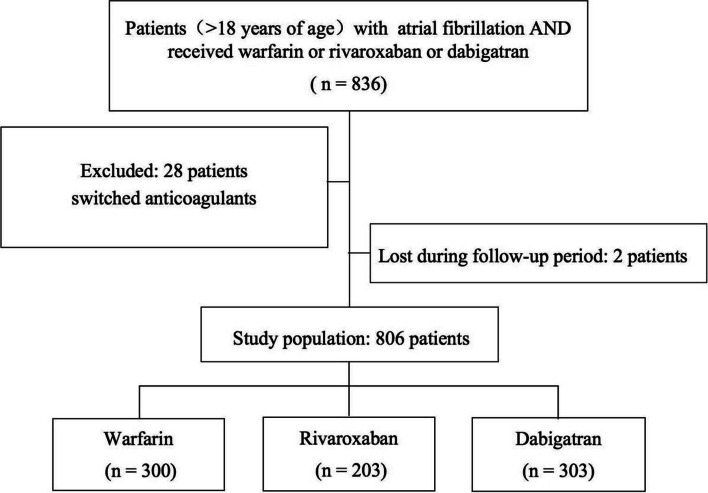
Study flow diagram describing patients population, inclusion and exclusion criteria for the retrospective cohort analysis

A total of 836 AF patients receiving anticoagulant therapy with warfarin, rivaroxaban or dabigatran were eligible for preliminary analysis. We excluded 28 patients for switching anticoagulants and 2 patients were lost during the follow-up period. Eventually, 806 patients were included: 300 patients received warfarin, 203 patients received rivaroxaban and 303 patients received dabigatran (Fig. 1).

Baseline characteristics of patients enrolled are shown in Table 1. The total average age of patients was 64.47: 62.82 for warfarin group, 65.81 for rivaroxaban group and 65.21 for dabigatran group. More than half of patients (57.3%) were male. The total average CHA2DS2-VASc score was 2.96: 2.86 for warfarin group, 3.02 for rivaroxaban group and 3.03 for dabigatran group. Numbers of patients with diabetes mellitus or stroke were similar in three groups. Most patients with hypertension and vascular diseases were in dabigatran group and most patients with heart failure were in warfarin group. Multivariate logistic regression analysis was conducted with bleeding events (Table 2) or IS events (Table 3) as dependent variables, and baseline characteristics such as age, hypertension, heart failure and vascular diseases as independent variables. Age was found to be an independent risk factor of bleeding (*P* = 0.010, OR = 0.969, 95% confidence interval 0.947—0.993).

**Table 1:** Baseline characteristics of the study population

Characteristic	Warfarin(*n* = 300)	Rivaroxaban(*n* = 203)	Dabigatran(*n* = 303)	Total (*n* = 806)	*P*		*P*	
						W vs R	W vs D	R vs D
Age(years)	62.82 ± 10.23	65.81 ± 10.92	65.21 ± 10.93	64.47 ± 10.74	0.003	0.007	0.006	0.773
Male(%)	163(54.3)	117(57.6)	182(60.1)	462(57.3)	0.361	0.465	0.155	0.586
Hypertension(%)	109(36.3)	102(50.2)	158(52.1)	369(45.78)	< 0.001	0.002	< 0.001	0.675
Diabetes Mellitus(%)	50(16.7)	43(21.2)	64(21.1)	157(19.5)	0.312	0.201	0.162	0.987
Stroke(%)	53(17.7)	39(19.2)	60(19.8)	152(18.9)	0.795	0.660	0.502	0.870
Heart Failure(%)	199(66.3)	53(26.1)	106(35.0)	358(44.4)	< 0.001	< 0.001	< 0.001	0.035
Vascular Disease(%)	137(45.7)	128(63.1)	178(58.7)	443(54.96%)	< 0.001	< 0.001	0.001	0.331
CHA2DS2-VASc Score	2.86 ± 1.48	3.02 ± 1.81	3.03 ± 1.70	2.96 ± 1.65	0.410	0.289	0.209	0.966
Smoking(%)	79(26.3)	37(18.2)	85(28.1)	201(24.9)	0.036	0.034	0.635	0.011
Alcohol Use(%)	51(17%)	25(12.3)	50(16.5)	126(15.6)	0.318	0.150	0.870	0.194
LDL-C, mmol/L	2.53 ± 0.87	2.36 ± 0.74	2.41 ± 0.83	2.44 ± 0.83	0.053	0.022	0.083	0.477
HDL-C, mmol/L	1.10 ± 0.34	1.10 ± 0.26	1.12 ± 0.28	1.11 ± 0.30	0.699	0.990	0.475	0.451
Triglyceride, mmol/L	1.49 ± 0.73	1.71 ± 1.14	1.50 ± 0.79	1.55 ± 0.87	0.014	0.01	0.789	0.021
Lipoprotein A, g/L	1.09 ± 0.27	1.18 ± 0.27	1.15 ± 0.23	1.14 ± 0.26	0.001	0.001	0.004	0.317
Lipoprotein B, g/L	0.88 ± 0.28	0.82 ± 0.23	0.85 ± 0.25	0.85 ± 0.26	0.039	0.013	0.112	0.256
Uric Acid, μmol/L	399.74 ± 148.17	338.87 ± 123.27	363.72 ± 119.35	370.93 ± 113.88	< 0.001	< 0.001	0.001	0.025
Crcl, mL/min	98.30 ± 48.25	91.59 ± 33.69	92.34 ± 40.15	94.38 ± 42.01	0.122	0.087	0.101	0.828
LAD, mm	47.39 ± 10.66	40.21 ± 6.46	42.77 ± 6.97	43.86 ± 8.91	< 0.001	< 0.001	< 0.001	< 0.001
LVEF (%)	54.94 ± 10.85	58.17 ± 8.78	56.51 ± 9.98	56.34 ± 10.10	0.003	0.001	0.074	0.061
CHA2DS2-VASc Score (%)								
Score 0–1	50(16.7)	50(24.6)	60(19.8)	160(19.9)	0.020	0.028	0.319	0.197
Score 2–3	157(52.3)	68(33.5)	127(41.9)	352(43.7)	0.426	< 0.001	0.010	0.057
Score ≥ 4	93(31.0%)	85(41.9)	116(38.3)	294(36.4)	0.308	0.012	0.060	0.419

*Abbreviations*: *Stroke* previous stroke, transient ischemic attack or thromboembolism, *LDL-C* low-density lipoprotein cholesterol C, *HDL-C* high-density lipoprotein cholesterol C, *LAD* left atrial diameter, *LVEF* left ventricular ejection fraction. W for warfarin, R for rivaroxaban, D for dabigatran. Data are expressed as mean ± SEM

**Table 2:** Multivariate logistic regression analysis of bleeding events

Variate	β	Se	Wald χ^2^	*P*	OR (95% Cl)
Age	-0.031	0.012	6.556	0.010	0.969 (0.947, 0.993)
Hypertension	-0.132	0.237	0.308	0.579	0.877 (0.551, 1.395)
Heart Failure	0.054	0.289	0.035	0.851	0.947 (0.538, 1.669)
Vascular Disease	-0.220	0.257	0.739	0.390	0.802 (0.485, 1.326)
Smoke	0.040	0.270	0.022	0.883	0.961 (0.566, 1.632)
Triglyceride	0.149	0.137	1.173	0.279	1.160 (0.887, 1.518)
Lipoprotein A	-0.336	0.534	0.395	0.530	0.715 (0.251, 2.038)
Lipoprotein B	-0.554	0.516	1.152	0.283	0.575 (0.209, 1.581)
Uric Acid	0	0.001	0.017	0.896	1.000 (0.998, 1.002)
LAD	0.007	0.015	0.209	0.648	1.007 (0.978, 1.036)
LVEF	0.026	0.015	3.026	0.082	1.026 (0.997, 1.057)
CHA2DS2-VASc Score (%)					
Score 0–1	0.629	0.527	1.424	0.233	1.876 (0.668, 5.71)

*Abbreviations*: *CI* confidence interval, *LAD* left atrial diameter, *LVEF* left ventricular ejection fraction

**Table 3:** Multivariate logistic regression analysis of IS events

Variate	β	Se	Wald χ^2^	*P*	OR (95% Cl)
Age	-0.002	0.022	0.008	0.927	0.998 (0.957, 1.041)
Hypertension	-0.400	0.421	0.903	0.342	0.670 (0.293, 1.530)
Heart Failure	-0.146	0.502	0.084	0.772	0.864 (0.323, 2.313)
Vascular Disease	0.335	0.443	0.573	0.449	1.399 (0.587, 3.334)
Smoke	0.115	0.471	0.060	0.807	1.122 (0.446, 2.825)
Triglyceride	-0.543	0.408	1.769	0.183	0.581 (0.261, 1.293)
Lipoprotein A	0.949	0.905	1.100	0.294	2.583 (0.439, 15.219)
Lipoprotein B	0.771	0.912	0.715	0.398	2.163 (0.362, 12.921)
Uric Acid	0.001	0.002	0.210	0.647	1.001 (0.997, 1.004)
LAD	0.010	0.025	0.163	0.687	1.010 (0.962, 1.060)
LVEF	-0.007	0.024	0.087	0.768	0.993 (0.948, 1.040)
CHA2DS2-VASc Score (%)					
Score 0–1	-0.254	0.858	0.087	0.767	0.776 (0.144, 4.171)

*Abbreviations*: *CI* confidence interval, *LAD* left atrial diameter, *LVEF* left ventricular ejection fraction

### Adherence

The adherence rate was calculated and shown in Table 4. The adherence rate of patients receiving warfarin is 56.7%, which is lower than either NOACs (*vs* 73.9% of rivaroxaban, *P* < 0.001; *vs* 73.6% of dabigatran, *P* < 0.001). We compared persistence of patients with different CHA2DS2-VASc scores and no difference was observed (score 0–1, 68.8%; score 2–3, 69.9%; score ≥ 4, 63.6%). In patients with score 0–1 or ≥ 4, there were a significant lower adherence rate in warfarin group (score 0–1, 50.0%; score ≥ 4, 47.3%) compared with rivaroxaban (score 0–1, 80.0%, *P* = 0.002; score ≥ 4, 67.1%, *P* = 0.008) and dabigatran (score 0–1, 75.0%, *P* = 0.007; score ≥ 4, 74.1%, *P* < 0.001) and a reduction in patients with score 2–3 (64.3% of warfarin, *vs* 77.9% of rivaroxaban, *P* = 0.045; *vs* 72.4% of dabigatran, *P* = 0.145). In warfarin-treated patients, those with moderate risk of stroke have higher persistence (score 2–3, 64.3%) than patients with low (score 0–1, 50.0%) or high (score ≥ 4, 47.3%) risk of stroke. Similar adherence rates were seen in rivaroxaban-treated and dabigatran-treated patients with different CHA2DS2-VASc scores.

**Table 4:** Adherence to OACs

CHA2DS2-VASc Score	Warfarin (*n* = 300)	Rivaroxaban (*n* = 203)	Dabigatran (*n* = 303)	Total (*n* = 806)	P		
					W vs R	W vs D	R vs D
All (%)	170(56.7)	150(73.9)	223(73.6)	543(67.4)	< 0.001	< 0.001	0.941
0–1(%)	25(50.0)	40(80.0)	45(75.0)	110(68.8)	0.002	0.007	0.533
2–3(%)	101(64.3)	53(77.9)	92(72.4)	246(69.9)	0.044	0.145	0.402
≥ 4(%)	44(47.3)	57(67.1)	86(74.1)	187(63.6)	0.008	< 0.001	0.274

*Abbreviations*: *W* for warfarin, *R* for rivaroxaban, *D* for dabigatran

### The safety outcomes

**Fig. 2:**
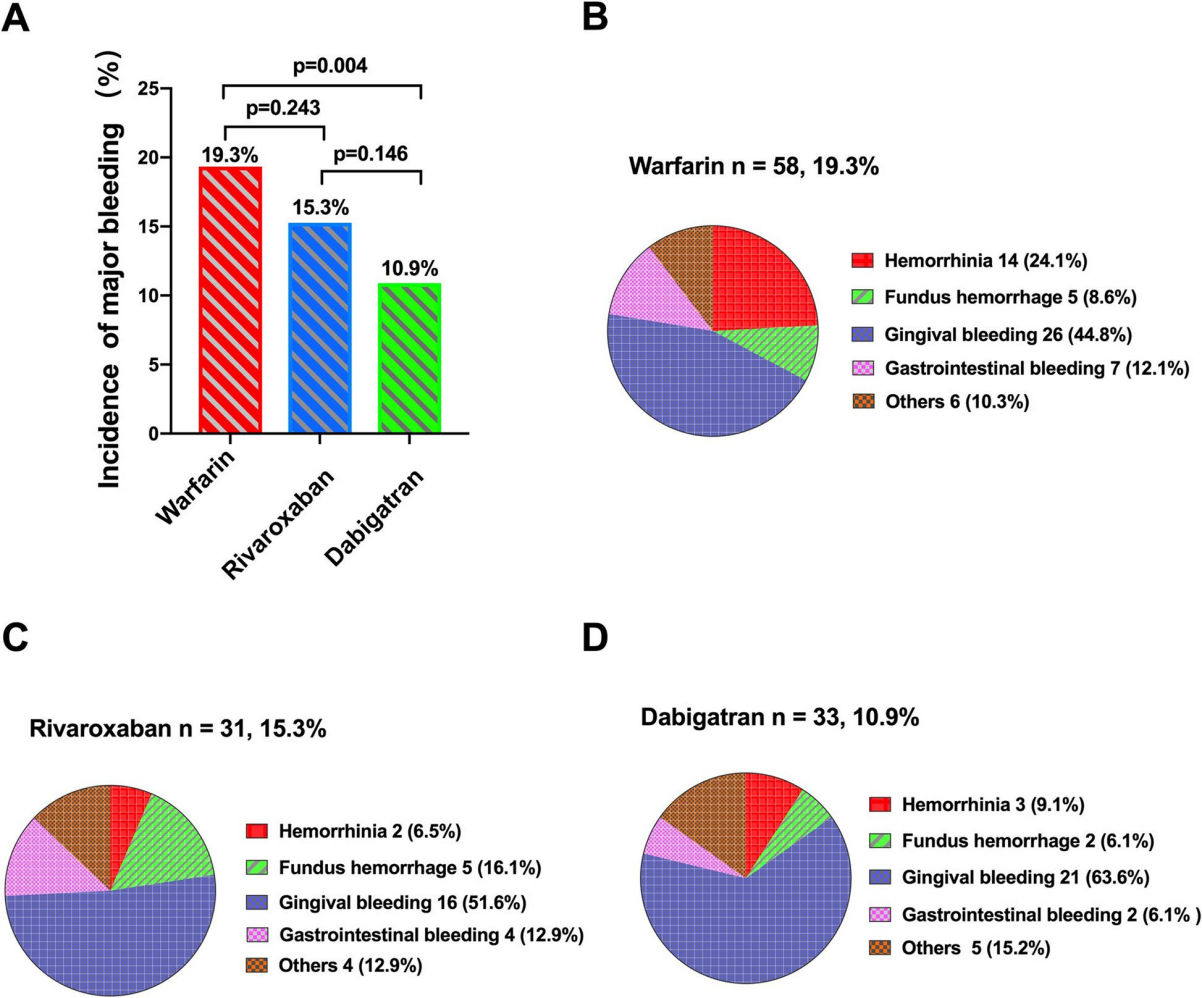
Safety outcomes. **A** Incidence of major bleeding; **B** Bleeding events distribution of warfarin therapy; **C** Bleeding events distribution of rivaroxaban therapy; **D** Bleeding events distribution of dabigatran therapy

The data regarding incidence of bleeding events were shown in Fig. 2. During the follow-up, 122 (15.1%) patients experienced bleeding events: 58 (19.3%) patients in warfarin group, 31 patients (15.3%) in rivaroxaban group and 33 patients (10.9%) in dabigatran group. Compared with warfarin group, there was a statistically significant and non-significant reduction of bleeding in dabigatran (χ^2^ = 8.385, *P* = 0.004) and rivaroxaban (χ^2^ = 1.372, *P* = 0.241) intervention respectively (Fig. 2A). There was 1 cerebral hemorrhage and 1 severe gastrointestinal bleeding event in patients receiving warfarin and 4 cerebral hemorrhage events in patients receiving rivaroxaban and no severe bleeding events in patients receiving dabigatran (data not shown). The most frequent bleeding events was gingival bleeding regardless of anticoagulants (Fig. 2B-D).

### The efficacy outcomes

**Fig. 3:**
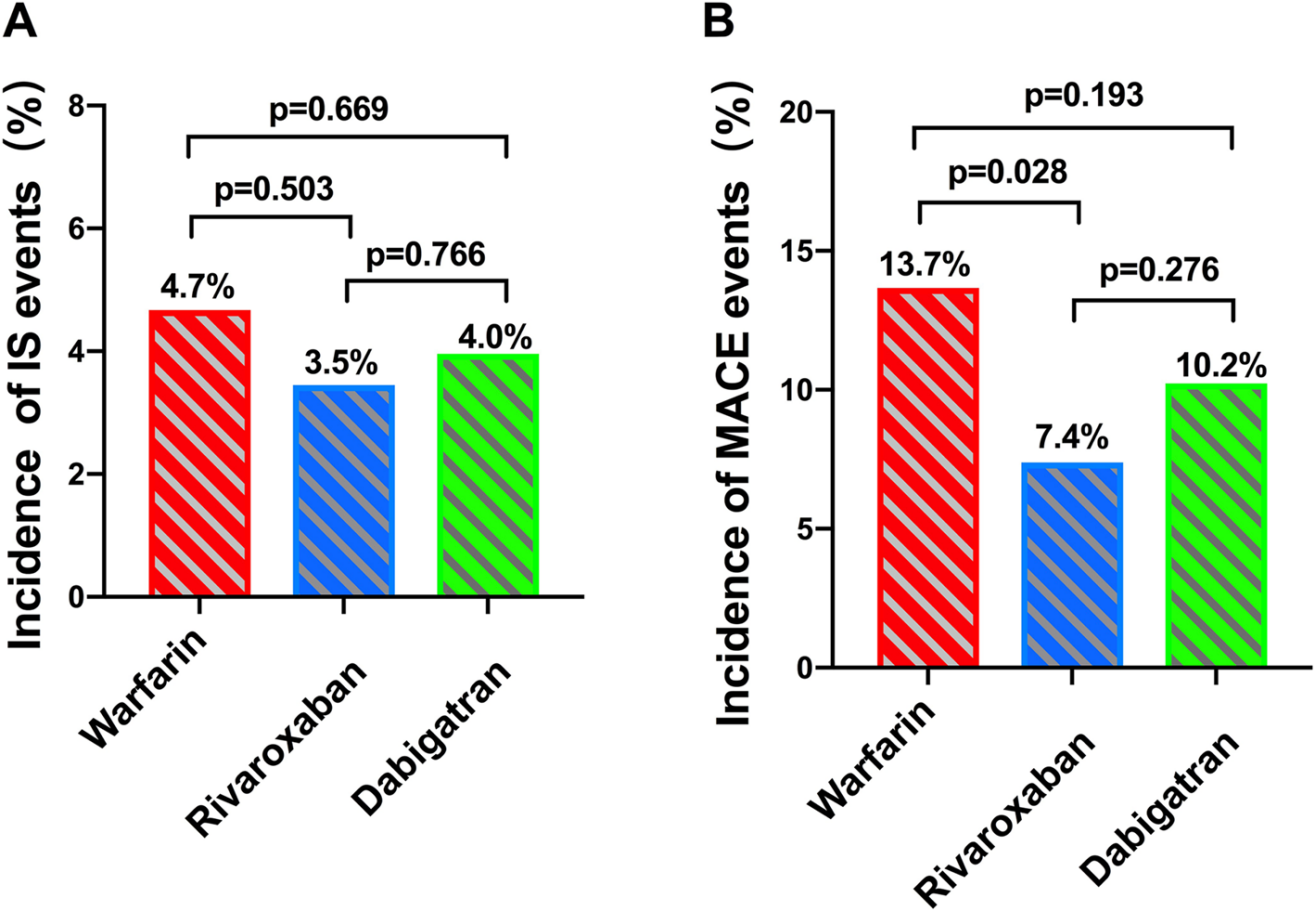
Efficacy outcomes. **A** Incidence of IS events; **B** Incidence of MACE events

During the follow-up, 33 (4.1%) patients experienced IS events, including 14 (4.7%) in warfarin group, 7 (3.5%) in rivaroxaban group and 12 (4.0%) in dabigatran group. Although more IS events were spotted in warfarin group, there were no statistically significant differences compared with NOACs (χ^2^ = 0.449, *P* = 0.503 *vs* rivaroxaban group; χ^2^ = 0.182, *P* = 0.669 *vs* dabigatran group) (Fig. 3A). Similarly, more MACE events were observed in warfarin group. 87 patients experienced MACE events: 41 patients (16.7%) in warfarin group, 15 patients (7.4%) in rivaroxaban group and 31 patients (10.2%) in dabigatran group. There was a significant reduction of MACE in rivaroxaban compared with warfarin therapy (χ^2^ = 4.822, *P* = 0.028) and a non-significant reduction with dabigatran (χ^2^ = 1.692, *P* = 0.193). Between the two NOACs, patients receiving rivaroxban experience fewer MACE albeit statistically not significant (χ^2^ = 1.188, *P* = 0.276) (Fig. 3B).

### Risks of bleeding according to CHA2DS2-VASc score

**Fig. 4:**
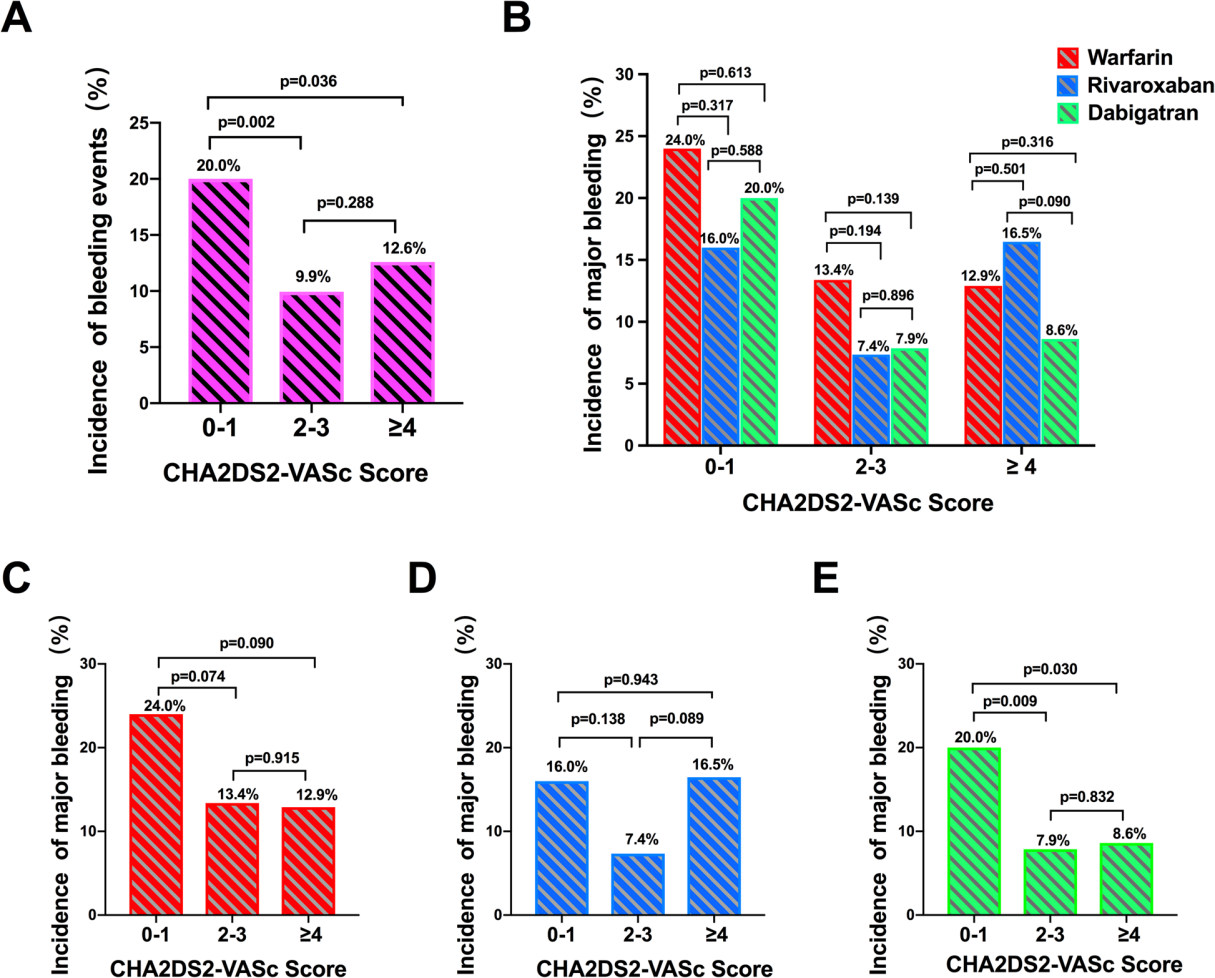
Incidence of bleeding events according to CHA2DS2-VASc scores with different OACs treatment. **A** Incidence of bleeding events according to CHA2DS2-VASc scores; **B** Major bleeding after different OACs treatment according to CHA2DS2-VASc scores; **C** Incidence of major bleeding after warfarin treatment; **D** Incidence of major bleeding after rivaroxaban treatment; **E** Incidence of major bleeding after dabigatran treatment

Patients were grouped into three according to CHA2DS2-VASc scores (score 0–1, 2–3, ≥ 4). Patients with score 0–1 had higher incidence rate of bleeding (20%) compared with patients of score 2–3 (9.9%, χ^2^ = 9.782, *P* = 0.002) and score ≥ 4 (12.6%, χ^2^ = 4.420, *P* = 0.036) (Fig. 4A). Incidence of bleeding did not differ significantly among patients within the same score group and receiving different anticoagulants. However, smaller incidence rate of bleeding was observed in rivaroxaban-treated patients with score 0–1 and 2–3, and dabigatran-treated patients with score ≥ 4 (Fig. 4B). In warfarin-treated patients, those with score 0–1 experienced more bleeding events than those with score 2–3 or ≥ 4 albeit statistically not different (Fig. 4C). In rivaroxban-treated patients, there was a non-significant reduction of bleeding in patients with score 2–3 compared with the other two groups, in which incidence rates of bleeding events were almost same (Fig. 4D). In dabigatran-treated group, patients with score 0–1 were more likely to experience bleeding (20.0%) than those with score 2–3 (7.9%, χ^2^ = 6.816, *P* = 0.009) or score ≥ 4 (8.6%, χ^2^ = 4.682, *P* = 0.030) (Fig. 4E).

### Risks of IS according to CHA2DS2-VASc score

**Fig. 5:**
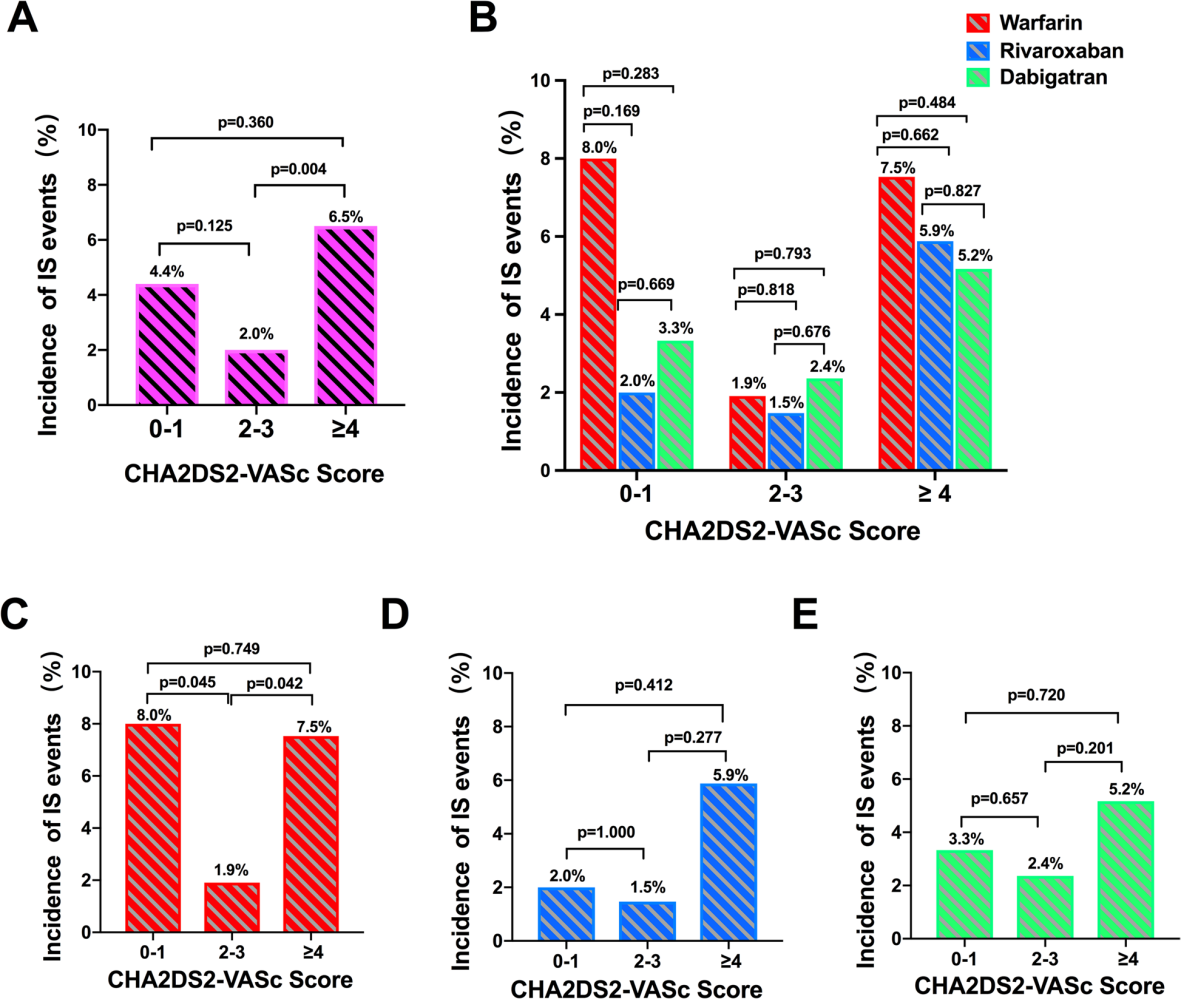
Incidence of IS events according to CHA2DS2-VASc scores with different OACs treatment. **A** Incidence of IS events according to CHA2DS2-VASc scores; **B** IS events after different OACs treatment according to CHA2DS2-VASc scores; **C** IS events after warfarin treatment; **D** IS events after rivaroxaban treatment; **E** IS events after dabigatran treatment

During the follow-up, 33 patients experienced IS events: 14 patients (4.4%) in score 0–1, 7 patients (2.0%) in score 2–3 and 12 patients (6.5%) in score ≥ 4. The incidence rate of IS in patients with score ≥ 4 is higher than that of patients with score 2–3 (χ^2^ = 8.301, *P* = 0.004) and not significant higher than patients with score 0–1 (χ^2^ = 2.355, *P* = 0.125) (Fig. 5A). Incidence of IS did not differ significantly among patients within the same score group and receiving different anticoagulants. However, smaller incidence rate of IS were spotted in rivaroxaban group in patients with score 0–1 and 2–3, and dabigatran group in patients with score ≥ 4 (Fig. 5B). In warfarin group, patients with score 2–3 have lower IS incidence rate (1.9%) than those with score 0–1 (8.0%, *P* = 0.045) and score ≥ 4 (7.5%, *P* = 0.042) (Fig. 5C). Incidence of IS were similar in patients receiving rivaroxaban and dabigatran with different CHA2DS2-VASc scores (Fig. 5D-E).

## Discussion

NOACs have emerged as promising alternatives to warfarin for comparable efficacy of preventing thromboembolic events and safety of decreasing major bleeding events. However, the conclusion may not be applicable to patients in China since OACs have concerns about increased risk of bleeding in Asian population [13, 14]. In this retrospective cohort study, we enrolled patients from a single medical center of northeast China to compare the efficacy and safety of warfarin, rivaroxaban and dabigatran in AF patients with different CHA2DS2-VASc scores. AF is the most frequent cardiac arrhythmia and one of the largest epidemics and public health challenges with increasing incidence and prevalence worldwide. Efforts are needed to reduce adverse outcomes associated with AF and to improve efficacy of preventing thrombotic events especially in China.

### Adherence

Adherence, an important facet of diseases remedy, is germane to clinical outcomes especially for patients with chronic illnesses requiring long-term treatment such as AF. Among the participants we enrolled, patients receiving warfarin had lower persistence than those receiving rivaroxaban or dabigatran. Although estimates of persistence may vary due to differences in study design, treatments and patients selection, et al, higher adherence of NOACs than VKA is common in multiple studies [15–17]. NOACs have their superority and inferiority to medication persistence compared with warfarin. NOACs don’t need complex monitoring or dose adjustment compared to warfarin but their price is high and indications are relatively narrow. In our study, COVID-19 is another factor influencing adherence. During the epidemic, patients’ visits to hospitals reduced due to difficulties in seeking medical treatment or concerns about being infected in medical institutions, leading to failure of getting new prescription of medications. Besides, patients’ awareness is non negligible since some patients included discontinued anticoagulants because they thought they were “cured” and didn’t need more medications. We compared adherence of patients with different CHA2DS2-VASc scores. Warfarin-treated patients with score ≥ 4 had smaller adherence rates compared with score 0–1 or 2–3, so did the rivaroxaban-treated patients albeit statistically not significant. What causes our concerns is that patients with score ≥ 4 are with high stroke risks and discontinuation of anticoagulant therapy is dangerous. Consistently, more IS events were seen in warfarin- or rivaroxaban-treated patients with score ≥ 4. More efforts are needed to improve medication persistence especially for high risk patients with warfarin therapy.

### Bleeding events

Bleeding is an intrinsic side effect of anticoagulant drugs. HAS-BLED [Hypertension, Abnormal renal/liver function, Stroke, Bleeding history or predisposition, Labile time in therapeutic range (INR), Elderly (> 65 years), Drugs/alcohol concomitantly] score is mostly used to predict bleeding risk in AF patients but high bleeding risk is not the contraindication of OACs since patients can benefit from anticoagulant therapy [12, 18]. In our study, patients receiving dabigatran are less prone to experience bleeding events than warfarin-treated patients which is consistent with the RE-LY trial–the first RCT studying dabigatran versus warfarin in AF patients and post-marketing observational data [19, 20].

There was a non-significant reduction of bleeding in rivaroxaban group compared with warfarin group. Gastrointestinal bleeding is reported to increase with NOACs than warfarin and concomitant treatment with proton pump inhibitors (PPI) is reported to reduce severe upper gastrointestinal bleeding in AF patients [21, 22]. In our study, gingival bleeding was the most frequent bleeding event among patients receiving OACs which called for more attention of patients education and self monitoring. This may be partly due to concomitant using of PPIs in some patients.

We compared bleeding of patients with different CHA2DS2-VASc scores, and patients with score 0–1 had higher incidence of bleeding than those with score 2–3 or ≥ 4, which warns us to cautiously condition the indication to thromboprophylaxis in patients with score 0–1. The sample size may affect experimental results, besides, new bleeding risk factors may be missed. During follow up, we focused on incidence of bleeding event but ignored new bleeding risk. For example, some patients may develop abnormal liver and/or kidney function after discharge, or combined using antiplatelet drugs due to other concomitant diseases which will increase the risk of bleeding in these patients. Patients at “low risk” according to CHA2DS2-VASc score (0 in men or 1 in women) are not recommended for antithrombolic medications since the bleeding risk is considered to outweigh potential benefits of thromboembolic risk reduction [23]. However, many of these “low-risk” patients still receive anticoagulants according to physicians’ prescription. Persistent or permanent AF, aging, and concerns about risk factors beyond CHA2DS2-VASc score are associated with physicians’ decision of OACs utilization. In our study, 160 patients (19.9%) with CHA2DS2-VASc score 0–1 received anticoagulants. Fox et al reported forty-four percent of patients from the Global Anticoagulant Registry in the FIELD-Atrial Fibrillation study with CHA2DS2-VASc score (0 in men or 1 in women) receiving OACs. The study showed thromboembolic and major bleeding events were rare regardless of OACs prescription [24]. However, one limitation of this study is that we didn’t distinguish CHA2DS2-VASc score of men and women respectively. Besides patients with “low risk”, patients with score 0–1 in our study include male patients with 1-score risk factor (*n* = 92) who should be considered for OACs according to AF guidelines. The incidence of bleeding of patients at “low risk” may be overestimated in our study. Nonetheless, risk-factor-based approach to stroke prevention may be more suitable than undue focus on “high-risk” patients. For AF patients at low thrombo-embolic risk, more studies about clinical decision-making are needed. In dabigatran-treated group, patients with CHA2DS2-VASc score of 0—1 were more likely to experience bleeding than those with score 2—3 or score ≥ 4 (*P* < 0.05). Results were similar in the other two OACs but without statistically significance. Studies about efficacy and safety of warfarin versus NOACs have been conducted but there is still a lack of head-to-head studies between two NOACs. We get the conclusion based on the current sample size and experimental settings. However, our research still has limitations and more efforts need to be made to verify the conclusion.

### Stroke prevention

We found no significant difference of stroke events in patients with different anticoagulants, but patients receiving NOACs were less prone to experience IS events than warfarin. Incidence of MACE was lower in rivaroxaban than dabigatran group. Our results inclined the efficacy of NOACs especially rivaroxban over warfarin. NOACs have been proved with comparable or better effects on stroke prevention than warfarin [19, 25, 26]. Large RCTs on NOACs’ head-to-head trial comparing efficacy and safety of NOACs are lacking so in guidelines no preference of NOACs is recommended. As the most world-wide used anticoagulants, warfarin does have definite anti-coagulation and stroke prevention effects in AF patients. What mainly limited warfarin is the narrow therapeutic interval. Warfarin is susceptible to numerous factors including genetics, concomitant drugs and food. Patients receiving warfarin need routine INR monitoring and dose adjustment. Adequate TTR (usually > 70%) is the foundation of warfarin’s efficacy and safety. In this study, we included patients with TTR of more than 80% during the whole follow-up period. Insufficient anticoagulation intensity of warfarin is partially due to insufficient INR monitoring, which is also one of the reasons for over-anticoagulation even bleeding [27–30].

AF is an independent but not the only risk factor of stroke. Main clinical stroke factors were identified through RCTs or cohort studies. Risk stratification models for stroke prediction in AF were constructed such as ABC-stroke (age, biomarkers, clinical history), CHADS2 (CHF history, hypertension history, age ≥ 75, diabetes mellitus history, stroke or TIA symptoms previously), and Anticoagulation and risk factors in atrial fibrillation (ATRIA) score [31–33]. CHA2DS2-VASc score was firstly proposed in 2010 by experts of University of Birmingham Centre for Cardiovascular Sciences, and has been mostly used and recommended in clinical guidelines thereafter [34–36]. Consistently, in our study, more stroke events were observed in patients with score ≥ 4. The incidence of IS in patients with score 0–1 was higher than those with score 2–3 notwithstanding statistically not different. Unbalance patients number may partly contribute to this result because actual stroke events of both groups were the same (*n* = 7) but patients with score 2–3 (*n* = 352) were more than two-fold of patients with score 0–1 (*n* = 160).

## Conclusions

Patients receiving NOACs have better persistence than warfarin users. There was no significant difference of adherence between patients with rivaroxaban or dabigatran therapy. Of all patients enrolled, patients with CHA2DS2-VASc score 0–1 are associated with higher bleeding than those with score 2–3 or ≥ 4, specially patients receiving dabigatran. Thus, cautiously conditioning the indication to thromboprophylaxis is needed in northern Chinese AF patients with CHA2DS2-VASc score 0–1.

## Data Availability

The datasets used and/or analyzed during the current study are available from the corresponding author on reasonable request.
